# Development and evaluation of the accuracy of an indicator of the appropriateness of interventional cardiology generated from a French registry

**DOI:** 10.1186/s13690-022-00885-4

**Published:** 2022-05-06

**Authors:** Florence Francis-Oliviero, Pierre Coste, Emilie Lesaine, Corinne Perez, François Casteigt, Jean-Marie Clerc, Nicolas Delarche, Akil Hassan, Bernard Larnaudie, Jean-Louis Leymarie, Louis-Rachid Salmi, Florence Saillour-Glenisson

**Affiliations:** 1 Inserm Bordeaux Population Health, U1219, ISPED, Univ Bordeaux, Bordeaux, France; 2 Medical Information Department, Bordeaux University Hospital, Bordeaux, France; 3 Hôpital Cardiologique-CHU de Bordeaux, Université de Bordeaux, Pessac, France; 4 Polyclinique Bordeaux Nord Aquitaine, Bordeaux, France; 5 Centre Hospitalier de Périgueux, Périgueux, France; 6 Centre Hospitalier de Pau, Pau, France; 7 Centre Hospitalier de Mont de Marsan, Mont de Marsan, France; 8 Clinique Saint Augustin, Bordeaux, France

**Keywords:** Angiography, Angioplasty, Practice registry, Appropriateness

## Abstract

**Background:**

Development of appropriateness indicators of medical interventions has become a major quality-of-care issue, especially in the domain of interventional cardiology (IC). The objective of this study was to develop and evaluate the accuracy of an indicator of the appropriateness of interventional cardiology acts (invasive coronary angiographies (ICA) and percutaneous coronary interventions (PCI)) in patients with coronary stable disease and silent ischemia, automated from a French registry.

**Methods:**

All ICA and PCI recorded in a Regional IC Registry (ACIRA) and operated for a stable coronary artery disease or silent ischemia from January 1st to December 31th 2013 in eight IC hospitals of Aquitaine, southwestern France, were included.

The indicator was developed to reflect European guidelines. Classification of appropriateness by the indicator, measured on the registry database, was compared to the classification of a reference standard (expert judgment applied through complete record review) on a random sample of 300 interventions.

Accuracy parameters were estimated. A second version of the indicator was defined, based on the analysis of false negative and positive results, and its accuracy estimated.

**Results:**

The second indicator accuracy was: sensitivity 63.5% (95% confidence interval CI [51.7–75.3]), specificity 76.0% (95%CI [70.4–81.6]), PPV 43.0% (95% CI [33.0–53.0]) and NPV 88.0% (95% CI [83.4–92.6]). When stratified on the type of act, parameters were better for ICA alone than for PCI.

**Conclusions:**

Accuracy of the indicator should raise with improvement of database quality. Despite its average accuracy, it is already used as a benchmark indicator for cardiologists. It is sent annually to each IC center with value of the indicator at the region level to allow a comparison.

## Background

All health systems face challenges in delivering high-quality, effective and safe care at an affordable cost. Improving appropriateness of care, *i.e.* the adequacy of care to patient needs in accordance with practice guidelines, is a major challenge, both for clinical care and public health policy [1].

Appropriateness of care has become of particular importance in the field of interventional cardiology (IC) [2]. IC includes all catheter-based procedures for treating congenital heart defects, rhythm disorders, or diagnosing and treating acute or chronic coronary artery disease. The latter indication of myocardial revascularization involves mainly two procedures: invasive coronary angiography (ICA) and percutaneous coronary intervention (PCI). These are invasive and costly interventions, potentially exposing patients to adverse events such as hematoma, arterial dissection, stroke, or related to toxicity of the contrast agent.

In 2014, major variations in standardized proportions of myocardial revascularization, from 111 to 371 procedures per 100 000 inhabitants, were observed in 12 Organization for Economic Co-operation and Development countries [3]; France, with 213 PCI per 100 000 inhabitants, was at the 8th position. One possible explanation for these variations relates to the appropriateness of IC and their consequences on the quality and safety of care. In the United States, appropriateness of PCI is now one of the inpatient quality indicators measured by the Agency for Healthcare Research and Quality [4].

Using the appropriate use criteria (AUC) for coronary revascularization and diagnostic catheterization, developed by the American College of Cardiology and five other professional organizations, recent studies showed higher proportions of inappropriate ICA and PCI in the non-acute context [5, 6]. For example, Chan et al. identified inappropriate use in 11.6% of non-acute PCIs in the United States National Cardiovascular Data Registry [7]. Bradley et al. also identified, in Washington State, inappropriate use in 17.0% in non-acute against 1.0% in acute PCIs [7]. In New York, Hannan et al. reported a proportion of 24.9% inappropriate ICAs [8]. Less information is available in Europe, and particularly in France, on the frequency of inappropriate ICA and PCI and no indicator of appropriate use has been implemented.

USA, Sweden, Germany, Great Britain, Denmark, Austria, and Japan have implemented regional or national IC registries, recording IC activity, and assessing medical practice to improve quality of care. From the registry databases, indicators may be measured to assess and monitor continuously the level of PCI or ICA appropriateness and to provide feedbacks [9, 10]. Implementation modalities vary between registries [11–13] and most of them only concern PCI. In France, such cardiovascular registries collect exhaustive and prospective data concerning interventional cardiology acts [14, 15]. One of them, the ACIRA registry is running since 2012 and includes all acts of interventional cardiology performed in the Aquitaine region, southwestern France [16].

No appropriateness indicators, to our knowledge, have been yet developed in France. Indeed, the AUC cannot directly be derived from European IC registries for two reasons: i) the criteria were developed from American guidelines for the management of stable coronary artery disease (SCAD) and can hardly be transposed to European guidelines that were develop by European experts considering the specificities of European health care systems; ii) they are based on clinical symptoms and intensity of medical treatment, data that are not available in European interventional cardiology registries information systems, particularly in France.

The objective of this study was to develop and evaluate the accuracy of an indicator of the appropriateness of ICA and PCI in patients with coronary stable disease and silent ischemia, automated from the ACIRA registry.

## Methods

### Study design

**Fig. 1:**
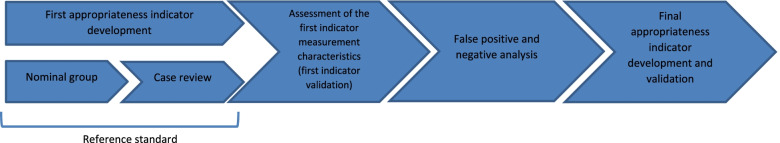
Stages of the development and validation of the appropriateness indicator

A first version of the indicator has been developed from the ACIRA database (Fig. 1). Its accuracy (sensitivity, specificity, positive predictive value (PPV), negative predictive value (NPV)) was estimated by comparing, on a random sample of interventions from the ACIRA database, the classification of appropriateness by the indicator and a reference standard, based on experts’ judgment. Reasons for false positives (FP) and negatives (FN) results were then investigated to construct a final indicator and improve its accuracy.

### The ACIRA registry

ACIRA is an exhaustive nominative and prospective cohort of patients cared with PCI, ICA or both. Included patients are all adults (≥ 18 years) French residents who underwent ICA or PCI in one of the 11 interventional cardiology hospitals (public university or local and private hospitals) in the Aquitaine region (southwestern France) and agreed to participate [16]. ACIRA registry identifies and describes patients who had an ICA or PCI in Aquitaine, assesses one-year outcomes and IC practices, healthcare pathway, and organization. It is meant to help cardiologists, researchers, healthcare decision-makers and hospitals answer questions about quality, safety, suitability, efficiency of interventional cardiology in the region and facilitate clinical and interventional research projects. ACIRA data are only extracted from existing databases. The data related to hospitalization and procedure is directly extracted from hospital information systems. Readmissions, in-hospital complications, cardiovascular morbidity and in-hospital mortality are collected from the French hospital medical information system database (PMSI), during one year after the initial procedure. An audit process of extractions checking, data harmonization and quality controls are carried out. Each year ACIRA includes about 15 000 IC acts.

### Study population and sample

The study population included all IC acts, registered in the ACIRA registry database, performed for the treatment or diagnosis of SCAD or silent ischemia (SI), from January 1st to December 31th 2013, in eight of the eleven interventional cardiology centers in the region. At the time of the study, three of the eleven centers had not yet implemented data extraction from their hospital information system. To estimate accuracy of the indicator, a sample of 300 interventions was randomly extracted from the study population. This threshold of 300 consisted in a fair balance between feasibility of data collection, and precision of the sensitivity and specificity estimations. Assuming a proportion of 30% inappropriate acts, estimated from a pilot study, 300 interventions were sufficient to estimate, with a two-sided 3% precision (confidence interval estimated with the normal law), an expected specificity of 95% and two-sided 7% precision around an expected sensitivity of 85%.

### Indicator development

The indicator was developed on the basis of the European Society of Cardiology guidelines [17, 18]. Only IA and IIIA grade recommendations have been considered (Table 1). Indicators were developed for each of three types of coronary disease management: ICA alone, ICA followed by PCI, and PCI alone. For ICA followed by PCI, ICA and PCI appropriateness were independently taken in consideration. If one of the two interventions was identified as inappropriate, the whole strategy was considered inappropriate.

**Table 1:** High graded 2014 ESC/EACTS Guidelines on myocardial revascularization for Interventional Coronary Angiography and Percutaneous Coronary Intervention indications

Guidelines issues	Recommendation	Class/Level
Indication of ICA	LVEF < 50% and either typical angina or PTP > 85% without prior non-invasive tests	IA
	Results of non-invasive testing were in favor of an ischemia and the PTP was between 15 and 85%	IA
Indication of revascularization for prognosis	A left main disease with stenosis > 50%,	IA
	Any proximal left anterior descending coronary artery stenosis > 50%	IA
	Two-vessel or three-vessel disease with stenosis > 50% with impaired LV function (LVEF < 40%)	IA
Indication of revascularization for symptoms	Any coronary stenosis > 50% in the presence of limiting angina or angina equivalent, unresponsive to medical therapy	IA
Non-indication of a PCI but recommendation of a CABG when a revascularization is recommended	Left main disease with a SYNTAX score > 32	IIIB
	Three-vessel disease with a SYNTAX score > 23	IIIB

*CABG* Coronary Artery Bypass Graft, *ICA* Interventional Coronary Angiography, *PCI* Percutaneous Coronary Intervention

As no data on symptoms or current treatment was available in the ACIRA database, all included patients with a SCAD were considered as having typical symptoms of stable angina and receiving optimal medical treatment. In the same way, calculation of the SYNTAX score has been partially calculated because of missing variables in the registry [19]. It is an anatomical prognostic score of severity, based on a precise description of the nature and complexity of the vascular lesions. Usually, it is calculated based on the vascular heart dominance, the localisation of segments that are diseased, the presence or absence of: total occlusion, trifurcations, bifurcations, aorto ostial lesion, severe tortuosity, heavy calcification and length of the disease superior to 20 mm. In the present study, using data from ACIRA registry, SYNTAX score has been calculated using the following items: stenosis grade, existence of trifurcations and number of segments diseased, and thrombus. Details concerning occlusion were not available in the ACIRA database and could not be implemented in the score calculation. In guidelines, when the SYNTAX Score is higher than 32 with a left main disease, or higher than 23 with a three-vessel disease, PCI is not recommended. Moreover, as the information about the dominance side could not be known from the ACIRA database, we have considered for the SYNTAX score calculation that all people had a right dominance.

Functional Flow Reserve (FFR) is a procedure performed during an ICA: if it was more than 0.80, it was considered that functional impact was insufficient to allow revascularization by PCI. Only the conduct of FFR was recorded in the ACIRA database, but not its results. If an FFR was done and all stenosis were less than 50%, and a revascularization followed, FFR was considered as being greater 0.8.

### Reference standard

The reference standard was based on experts’ judgment. It was conducted in two steps: regional consensus and case review. Experts were referent cardiologists from the registry centers, who accepted to participate in the process.

#### First step: regional consensus

The nominal group technique (NGT) was used to obtain a regional consensus on ICA and PCI indications [20]. The objectives of the NGT meeting were to define an exhaustive and unambiguous classification of any patient with stable angina or silent ischemia, allowing sorting them according to the appropriateness of the act they received. The NGT was organized in four steps: silent generation of ideas in response to the question, “round robin”, clarification, and voting. The five experts were asked the following question: “Which clinical and management elements do determine appropriateness or inappropriateness of ICA and PCI in the context of SCAD and SI?” A proposal was considered as consensual if four of five cardiologists had voted for it. Finally, consensually-retrieved items were organized by two of the authors (FF and FSG) to produce the appropriateness classification. This classification allowed carrying out the case review on a consensual basis.

#### Second step: case review

During the case review, the experts judged appropriateness of the acts randomized in the study sample. Information needed to assess appropriateness of interventions (clinical data such as medical history, symptoms, type of ischemia, radiological examinations and results…) was collected from patient record files and recorded on a standardized grid. Content of this grid had been previously validated by the cardiologists. To judge the appropriateness of the 300 randomly selected interventions, experts were asked to use the previously defined classification. Each case was independently assessed by two experts, each pair reviewed approximately 100 files. Experts assessed appropriateness blindly from each other, and did not know in which center the intervention had been done. If they disagreed, a third expert was asked to settle.

### Accuracy and discordance analyses

Sensitivity, specificity, PPV, and NPV of the indicators were estimated, using expert’s judgment as the reference standard. A true positive (TP) was an intervention defined inappropriate by both experts and indicator. Corresponding 95% confidence intervals (CI) were estimated using normal approximation [21]. Measurement characteristics were calculated first for all 300 interventions, then stratified for ICA alone, ICA followed by a PCI, and PCI alone.

Two experts (FC and JLL) analyzed extracted files of false positives (FP) and negatives (FN) results to understand mechanisms leading to discordances between the reference standard and the indicator. Then a new indicator (final indicator) was developed with an independent cardiologist, who was not involved in previous phases, and its accuracy was estimated.

We conducted robustness analyses on hypothesis made (approximation of Syntax score) by adding and subtracting 5 points to Syntax score calculated and by imputing data regarding eight files that had been excluded because experts were unable to decide on their appropriateness.

## Results

### Study population and sample

From the 15 233 interventions in the 2013 ACIRA database, 5 678 were selected in the study population (3 612 for SCAD and 2 066 of SI); 35.1% had a previous PCI, and 62.8% had high blood pressure. Clinical characteristics of the study sample are described in Table 2.

**Table 2:** Clinical characteristics of the randomly selected study sample for the validation of the appropriateness tool (*n* = 300)

	Sample		
	MV	N	%
Male gender		229	76.3
Ischemia type			
Stable angina		191	63.7
Silent ischemia		109	36.3
Antecedent of			
PCI	4	104	35.1
CABG	3	24	8.1
Myocardial infarctus	21	47	15.7
Stroke	3	10	3.4
LEVF < 50%	88	34	16.1
Chronic renal failure	4	8	2.7
Arteriopathy	4	38	12.8
Cardiovascular risk factors			
Current smoking	12	50	17.4
Diabetes	0	93	31.0
Dyslipidemia	4	191	65.0
Coronary heredity	10	61	21.0
Obesity (BMI > = 30 kg/m^2^)	17	68	24.0
Arterial hypertension	2	187	62.8
Positive non-invasive tests	0		
Electrocardiogram modifications		53	17.7
Elevated cardiac enzyme		11	3.7
Stress test		84	28.0
Myovardial scintigraphy		38	12.7
Stress echocardiography		35	11.7
Cardiac RMI		0	0.0
Coroscanner		3	1.0
FFR analysis	55	14	5.7

*CABG* Coronary Artery Bypass Graft, *FFR* Fractional flow reserve, *MV* missing values, *LVEF* Left Ventricular Ejection Fraction, *PCI* Percutaneous Coronary Intervention, *RMI* Resonance Magnetic Imaging

### Indicator development and accuracy

Among the 300 interventions, eight were excluded, for which experts did not succeed in assessing appropriateness, because of missing relevant clinical data. Among the remaining interventions, 88 (30.1%) were classified as inappropriate by the indicator, whereas 63 (21.6%) were judged as inappropriate by the experts (24.2% for ICA, and 11.2% for PCI). Sensitivity was 57.1% (95% CI [44.9–69.3]) and specificity 77.3% (95% CI [71.9–82.7%]) (Table 3).

**Table 3:** Diagnostic performances of the initial and final tool stratified according to the type of act (ICA only, ICA followed by PCI or PCI only)

Performances	First version	Final version			
	Total (*n* = 292)	Total (*n* = 284)	ICA only (*n* = 176)	ICA followed by a PCI (*n* = 43)	PCI only (*n* = 108)
	% (95% CI)		% (95% CI)		
Sensitivity	57.1 (44.9–69.3)	63.5 (51.7–75.3)	76.0 (64.2–87.8)	100.0 (69.2–100.0)	0.0 (0–30.9)
Specificity	77.3 (71.9–82.7)	76.0 (70.4–81.6)	73.8 (66.1–81.5)	57.5 (42.2–72.8)	87.3 (78.5–96.1)
PPV	40.9 (30.6–51.2)	43.0 (33.0–53.0)	53.5 (41.9–65.1)	46.5 (24.6–68.4)	0.0 (0–41.0)
NPV	86.8 (82.2–91.4)	88.0 (83.4–92.6)	88.6 (82.4–94.8)	53.5 (40.5–66.5)	82.3 (72.5–92.1)

*ICA* Invasive Coronary Angiography, *PCI* Percutaneous Coronary Intervention, *PPV* Positive Predictive Value, *NPV* Negative Predictive Value, *CI* Confidence interval

### False positive and negative results

The 52 FPs and 27 FNs were due to two main reasons. Firstly, missing values in the registry (probably not completed by cardiologists) yet mentioned in the medical files (especially concerning past history, results of tests and exams performed) for 33 (63.5%) FPs and 6 (22.2%) FNs. Secondly, a particular clinical context not taken into account in the indicator algorithm because of a lack of details in the registry, explained 12 (23.1%) FPs and 15 (55.6%) FNs. Particularly, the simplification made by considering only age and symptoms in the first step to evaluate appropriateness of ICA lead to misclassification. Four FPs occurred because of variation among cardiologists of the definition of SCAD in the ACIRA database. Some cardiologists considered as “stable angina” situations of acts performed in the month following an acute coronary syndrome, while others consider it as acute coronary syndrome. Lastly, approximations in the SYNTAX score calculation explained 3 (5.8%) FPs and 1 (3.2%) FN; 4 (7.7%) FPs and 5 (9.6%) FNs remained unexplained.

### Final indicator definition and accuracy

**Fig. 2:**
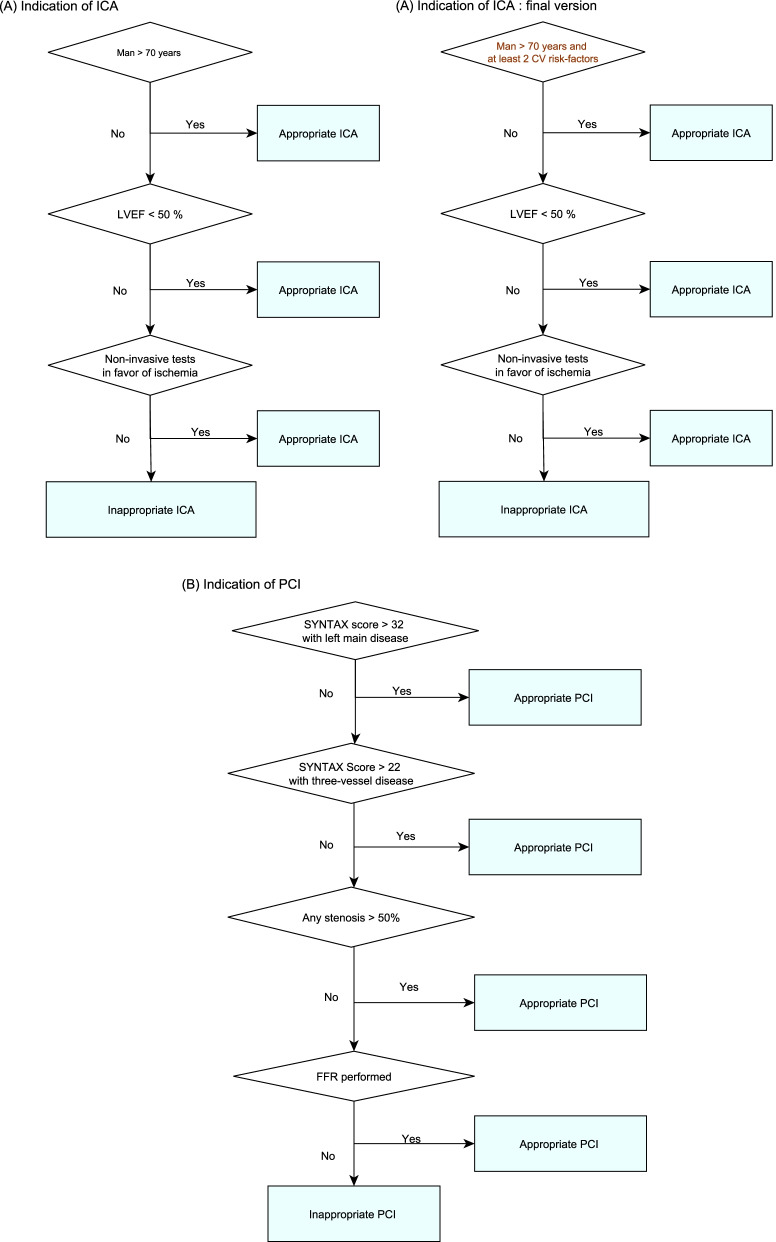
Presentation of the appropriateness indicators

Notion of severe coronary medical history (at least two cardiovascular risk factors among diabetes, hypertension and smoking) was added to age and sex to evaluate the pretest probability and indicate or not an ICA (Fig. 2). To avoid problems linked to stable angina diagnostic definition, patients with an acute coronary syndrome in the month before an interventional act (PCI or CAG) and a SCAD diagnosis in the database were excluded.

Final indicator sensitivity was 63.5% (95% CI [51.7–75.3]) and specificity was 76.0% (95%CI [70.4–81.6]). When stratified on the type of act (Table 3), parameters were better for ICA alone than for PCI.

### Robustness analysis (Table 4)

**Table 4:** Diagnostic performances after robustness analysis

Type of robustness analysis	Sensitivity	Specificity	PPV	NPV
	Estimate, % (95% CI)	Estimate, % (95% CI)	Estimate, % (95% CI)	Estimate, % (95% CI)
Addition of 5 points to the Syntax score	66.7 (55.1–78.3)	72.0 (66.1–77.9)	40.4 (31–49.8)	71.9 (65.3–78.5)
Subtraction of 5 points to the Syntax score	65.1 (53.3–76.9)	75.6 (69.9–81.3)	43.2 (33.2–53.2)	88.4 (83.8–93.0)
Addition of the 8 files, considering them all inappropriate	66.2 (55.2–77.2)	74.2 (68.4–80.0)	45.2 (35.6–54.8)	87.2 (82.4–92.0)
Addition of the 8 files, considering them all appropriate	65.1 (53.3–76.9)	72.5 (66.7–80.3)	39.4 (30.1–48.7)	88.3 (83.7–92.9)

*CI* confidence interval

When adding 5 points to the SYNTAX score, the indicator had a sensitivity of 66.7% (95%CI [55.1–78.3]), and specificity of 72.0% (95%CI [66.1–77.9]). When subtracting 5 points, sensitivity was 65.1% (95%CI [53.3–76.9]) and specificity 75.6% (95%CI [69.9–81.3]). When completing database records with information found in clinical files, all measurement parameters dramatically improved: sensitivity was 76.2% (95%CI [65.7–86.7]), specificity 89.6% (95%CI [84.7–93.6]), PPV 67.6% (95%CI [56.7–78.5]), and NPV of 93.0% (95%CI [84.7–89.5]).

## Discussion

We conducted the first study developing and validating an appropriateness indicator of PCI and ICA in a SCAD or SI context, from a French interventional cardiology database. A detailed analysis of FPs and FNs sources allowed an improvement of the indicator’s accuracy. From this perspective, final results suggest that the indicator might be improved through the improvement of ACIRA database completeness (in process). A new evaluation of indicator’s accuracy after improvement of database quality should be performed. Despite its average accuracy, it is already used as a benchmark indicator for cardiologists. Each center receives annually the value of the indicator at the center level compared to regional value. The idea is not to conclude on the basis of numerical results, but to have a reference value year after year, which allow cardiologists to question their practices.

The indicator had a very low sensitivity to detect inappropriate PCI, even in its final version, leading us not to consider it as a valid tool for the assessment of PCI inappropriateness. Analysis of FNs and FPs allowed us to understand the reasons of poor accuracy. Some hypotheses, guideline approximations and simplifications had to be done in the indicator construction, due to lack of information in the ACIRA database inappropriate. PCI were difficult to circumscribe with details from guidelines, which include many grade-II recommendations, difficult to implement into the tool, because of their uncertainty. Another reason of the low accuracy to detect inappropriate PCI is linked with the approximation made to calculate the SYNTAX score. This could explain the difficulty of the indicator to detect inappropriate PCIs; moreover, all unexplained cases after discordance analysis were PCI cases. This analysis allowed us also to precise and adapt our indicator to the database.

A robustness analysis assuming a perfectly completed database dramatically improved accuracy. Another element is concordant with filling limits of the ACIRA registry: all randomly selected records of our study sample concerned patients with SCAD or SI (except those secondarily excluded), but eight were recorded with only an enzyme elevation. Thus, the ability of our indicator to predict appropriateness could be improved by improving the database quality by cardiologists. Implementation of feedback and, more broadly, the use of this indicator could encourage cardiologists to improve the filling of the database. An advocacy for improvement and harmonization of ACIRA completion is ongoing and impact on quality of data will be further evaluated.

### Comparison with the literature

Proportions of inappropriate ICA and PCI, as estimated by experts, were comparable with previous reports [7, 8] However, a Swiss study showed a much higher proportion of inappropriate ICA in a stable clinical context (37.5%) [22]. These results could be explained by the differences in the guidelines used. The authors considered an ICA as inappropriate only when prior non-invasive tests had not been performed, whereas according to our guidelines, some ICA could be done in first line, if the medical history of the patient indicated a pre-test probability of cardiovascular disease superior to 85% (men > 70 years old and at least two cardio-vascular risk factors, in the final indicator).

### Strength of the study

The reference standard consisted in a case review by experts, after the implementation of a NGT to improve judgment homogeneity and consensus among experts. The request of a third expert when necessary limited the classification bias regarding the definition of the reference test. The NGT has been implemented using methodological standards [20]. Attention has been paid to the independence and blindness of the experts during the case review. Expressing a consensus of good practices during the NGT allowed us recording relevant variables for the review. Efforts made through the NGT to elicit appropriate indications also reinforced validity of the reference test.

The important involvement of interventional cardiologists from Aquitaine centers was another strong component. They were representative of all types of centers, private, public, and from all around the region. Their involvement in this project makes us confident that the development of such indicators is expected to fuel discussion on clinical practices not only in Aquitaine but also elsewhere in France.

### Limits

The low accuracy of detection of inappropriate PCI is one of the major limits. Moreover, hypotheses made led to decrease indicator’s accuracy. For example, all patients diagnosed with a stable angina were considered as having typical angina symptoms and being under optimal medical therapy. We assumed this happens for a large majority of patients undergoing an IC act. We plan to conduct an ad hoc study to evaluate and collect data on symptoms and treatments to confirm this hypothesis. This may have led to underestimate the proportion of inappropriate PCI, which was still higher than expected.

A lack of precision in estimations of measurement characteristics occurred because our first hypotheses on expected values on specificity and sensitivity were too optimistic. We have limited incorporation bias, which occurs when the reference standard includes the index test, by developing the reference standard independently from the indicator: experts were blinded to the indicator during the NGT and were judging appropriateness of the cases review independently of each other. Moreover, the final indicator has been developed with an independent expert. During case review, the experts knew ICA intervention results of each patient case, which could lead to an information bias. Some cardiologists may have been influenced by these results in their judgment of ICA appropriateness and may have overestimated ICA inappropriateness in case of normal ICA results. Finally, although allowing us to better target the patients to be included in the algorithm, the exclusion of the eight records corresponding to the acute context might have overestimated the accuracy of the final indicator. These eight cases had to be excluded during the review process, because of missing relevant data preventing the experts to assess appropriateness. Once the review sessions were carried out, it was not possible to include a posteriori eight new cases to compensate for the missing data. Notably due the difficulty to re-ask cardiologists to review another batch of 8 files, given their availabilities.

## Conclusion

We developed and validated an ICA appropriateness indicator whom accuracy should improve with the improvement of ACIRA database completeness. Indeed, this study highlighted for investigators and cardiologists, the value of completing the registry to develop more complete and accurate indicators.

Developing such a tool appears as an opportunity for cardiologists to benchmark with other cardiologists at a national and international level; it could also reduce inappropriate acts that expose patients to discomfort, avoidable hospitalizations or complications, and represent heavy costs for healthcare system. By testing its transferability, the indicator could be proposed to other European interventional cardiology registries constituted according to the same scheme and variables. Another perspective is to determine structural, organizational and individual factors associated with inappropriate IC. In summary, developing and validating such computerized quality indicator allowed us to confirm the great interest shown by cardiologists to evaluate and improve their clinical practices and reduce the proportion of inappropriate acts.

## Data Availability

The data that support the findings of this study are available from Dr Emilie Lesaine but restrictions apply to the availability of these data, which are not publicly available. Data are however available from the authors upon reasonable request and with permission of Dr Emilie Lesaine.

## Abbreviations

AUC: Appropriate Use Criteria
CABG: Coronary Artery Bypass Graft
ESC: European Society of Cardiology
FFR: Functional Flow Reserve
FN: False Negative
FP: False Positive
TN: True Negative
TP: True Positive
ICA: Invasive Coronary Angiography
NGT: Nominal Group Technique
NPV: Negative Predictive Value
PCI: Percutaneous Coronary Intervention
PPV: Positive Predictive Value
SCAD: Stable Coronary Artery Disease
